# Intramuscular hematoma of the vastus lateralis following percutaneous skeletal muscle microbiopsy: a case report

**DOI:** 10.14814/phy2.15038

**Published:** 2021-10-11

**Authors:** Patrick J. Drouin, Hashim Islam, Craig A. Simpson, Brendon J. Gurd

**Affiliations:** ^1^ School of Kinesiology and Health Studies Queen’s University Kingston Ontario Canada; ^2^ School of Medicine Queen’s University Kingston Ontario Canada

**Keywords:** case report, hemorrhage, intramuscular hematoma, recovery, risk mitigation, skeletal muscle biopsy

## Abstract

Recently, percutaneous microbiopsy needles have been used as a less invasive alternative to the Bergstrom needle for obtaining human skeletal muscle biopsy to assess changes in protein content, gene expression, and enzymatic activities. Unlike the Bergstrom muscle biopsy procedure, potential complications associated with microbiopsies of human skeletal muscle have not been documented. Therefore, the present case report follows a young male's recovery from a muscle biopsy‐induced hemorrhage/hematoma of the right vastus lateralis with the specific aims of (1) informing future participants, researchers, and clinicians on expected time course of recovery and (2) informing methods to minimize future participant adverse event risk during and after the percutaneous microbiopsy procedure. The present case report demonstrates that the inadvertent hemorrhaging of a neighboring vessel by percutaneous microbiopsy procedure can be debilitating. To minimize the risk of muscle biopsy‐induced hemorrhage/hematoma, we advise post‐biopsy compression for up to 15 min and post‐biopsy follow‐up should be completed for up to 72 h. When there is indication of hematoma development, compression should be applied, and the participant should avoid exercise and physical activity.

## Graphical abstract

The present case report demonstrates that the inadvertent hemorrhaging of a neighboring vessel by percutaneous microbiopsy procedure can be debilitating. To minimize the risk of muscle biopsy‐induced hemorrhage/hematoma, post biopsy compression is recommended for up to 15 min and post biopsy follow‐up should be completed for up to 72 h.

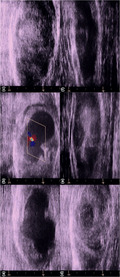

## 1 INTRODUCTION

Skeletal muscle biopsies are commonly completed to assess changes in protein content (Egan et al., 2013; Little et al., 2010), gene expression (Islam et al., 2019; Mahoney et al., 2005), and enzymatic activities (Carter et al., 2001; MacDougall et al., 1998) in response to exercise. The procedure is typically performed using the Bergstrom needle biopsy technique (Ekblom, 2017; Shanely et al., 2014), which is relatively less invasive compared to the open biopsy method and has a minimal complication rate (see Tarnopolsky et al. (2011) for detailed statistics). Recently, percutaneous microbiopsy needles have been used as an alternative to the Bergstrom needle for obtaining human skeletal muscle biopsy (Hughes et al., 2015). Although the microbiopsy technique results in the extraction of a smaller piece of muscle tissue than the Bergstrom needle, it does not require a pre‐biopsy incision of the skin, involves a lower gauge needle and is perceived as being less invasive and painful than the Bergstrom technique (Bonafiglia et al., 2020; Hughes et al., 2015).

Unlike the Bergstrom muscle biopsy procedure, potential complications associated with microbiopsies of human skeletal muscle have not been documented. Further, due to the infrequency of complications associated with Bergstrom needle muscle biopsies (Tarnopolsky et al., 2011) there is a deficiency of information regarding the development and progression of complications as well as the lived experiences of affected individuals. Therefore, the present case report follows a young male's recovery from a percutaneous muscle biopsy‐induced hemorrhage/hematoma of the right vastus lateralis with the specific aims of (1) informing future participants, researchers, and clinicians on expected time course of recovery and (2) informing methods to minimize future participant adverse event risk during and after the microbiopsy procedure.

## 2 METHODS

### 2.1 Participant characteristics

A 23‐year‐old healthy, non‐medicated, non‐smoking, and active Caucasian male (height: 187 cm; weight: 75.4 kg) undertaking ~3 h of moderate‐to‐vigorous physical activity per week.

### 2.2 Muscle biopsy procedure

A single muscle biopsy was obtained from the lateral portion of the right vastus lateralis at the midpoint between the anterior spina iliaca superior and the patella via the percutaneous microbiopsy technique (Hughes et al., 2015) using a 14‐gauge Medax Biofeather microbiopsy disposable needle. The skin was punctured under local anesthesia (2% xylocaine with epinephrine) perpendicular to horizontal using a 12‐gauge cannula inserted 4 cm deep into the muscle to guide the biopsy needle to a final depth of 8 cm. Three cuts (~10–20 mg each) were made with a ~90° rotation applied to the needle between each cut over a ~30 s period. Each piece of muscle was removed from the needle using a sterile disposable surgical blade before a subsequent cut was made.

### 2.3 Pain measurement

Pain was characterized using the short‐form McGill pain questionnaire (SF‐MPQ) completed 5, 6, 7, 8, 12, 13, and 19 days following the muscle biopsy (Melzack, 1987). Overall pain intensity was quantified using the present pain inventory (PPI) and a visual analog scale (VAS), included in the SF‐MPQ.

### 2.4 Hematoma imaging

A PhD candidate with ultrasound expertise recorded transverse ultrasound images of the affected site using a linear echo ultrasound probe operating at 13‐MHz in 2D mode (Vivid‐I GE Medical Systems).

## 3 CASE PRESENTATION

### 3.1 Unconfirmed pulsatile period

*Day 0*. The muscle biopsy was performed on the participants right leg at 08:30 on Day 0. Immediately post‐biopsy, the participant experienced stiffness and favored his right leg. In the afternoon, the participant experienced pain while flexing his leg and had difficulty jumping while playing volleyball.

*Day 1*. The following day leg pain did not improve and the participant demonstrated an antalgic gait pattern. At 20:00 of the same evening, after having been seated for approximately 2 h, the participant stood up and experienced immediate severe sharp, shooting, and throbbing pain. Visual inspection of the limb revealed slight bleeding, swelling, and warmth in the area surrounding the biopsy site. The participant contacted the primary investigator and was prescribed rest, ice, compression, and elevation. Before bed, the participant took 800 mg ibuprofen—severe sharp pain persisted making sleep difficult.

*Day 2*–*4*. Pain in the participant's right leg was reduced to moderate. The participant maintained an antalgic gait pattern and reported moderate shooting pain throughout the entire length of his right leg upon transition from sitting to standing. On day 4 the participant assisted with moving furniture for ~4‐h. Severity of pain remained moderate throughout day 4, and in the evening tightness of the leg had increased.

### 3.2 Confirmed pulsatile period

**FIGURE 1:**
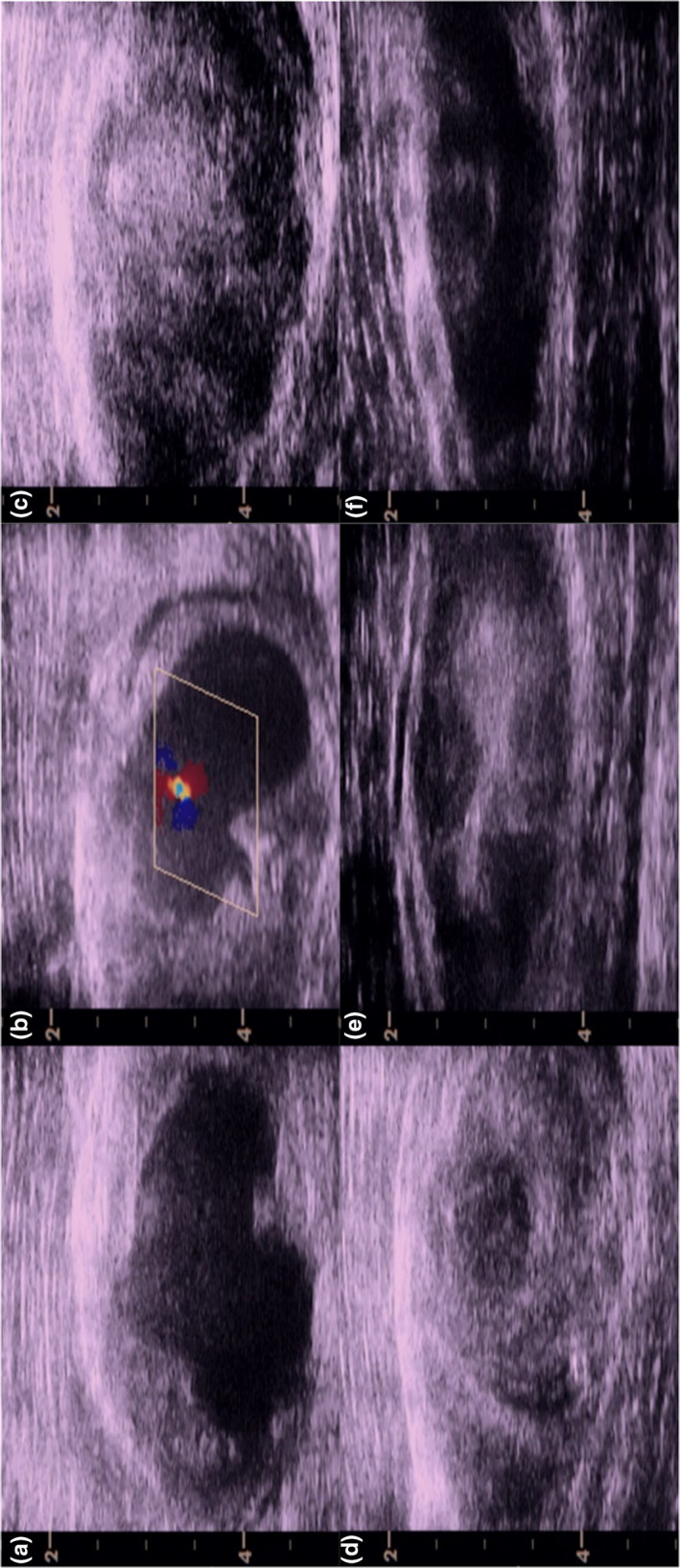
Transverse ultrasound images of right vastus lateralis showing the intramuscular hematoma five (a), six (b), eight (c), nine (d), twelve (e), and nineteen (f) days after percutaneous microbiopsy. Color in (b) reflects arterial flow in the heterogeneous mass. Y‐scale is measured in cm

*Day 5*. On day 5 the participant reported a recurrence of the severe sharp, shooting, and throbbing pain experienced on day 1. The participant could not walk or bend his leg without severe pain. The participant was driven to the School of Kinesiology and Health Studies where an ultrasound of the anterolateral aspect of the right thigh was completed. Although a pulsatile image was not saved, the ultrasound revealed a heterogeneous mass with arterial inflow in the deeper aspect of the right vastus lateralis, below the site of incision (Figure 1a). The participant rested, began compression, iced his leg and took 800 mg ibuprofen. The participant was not instructed to abstain from strenuous exercise, though the participant reported that he was unable to complete even low‐moderate exercise.

**FIGURE 2:**
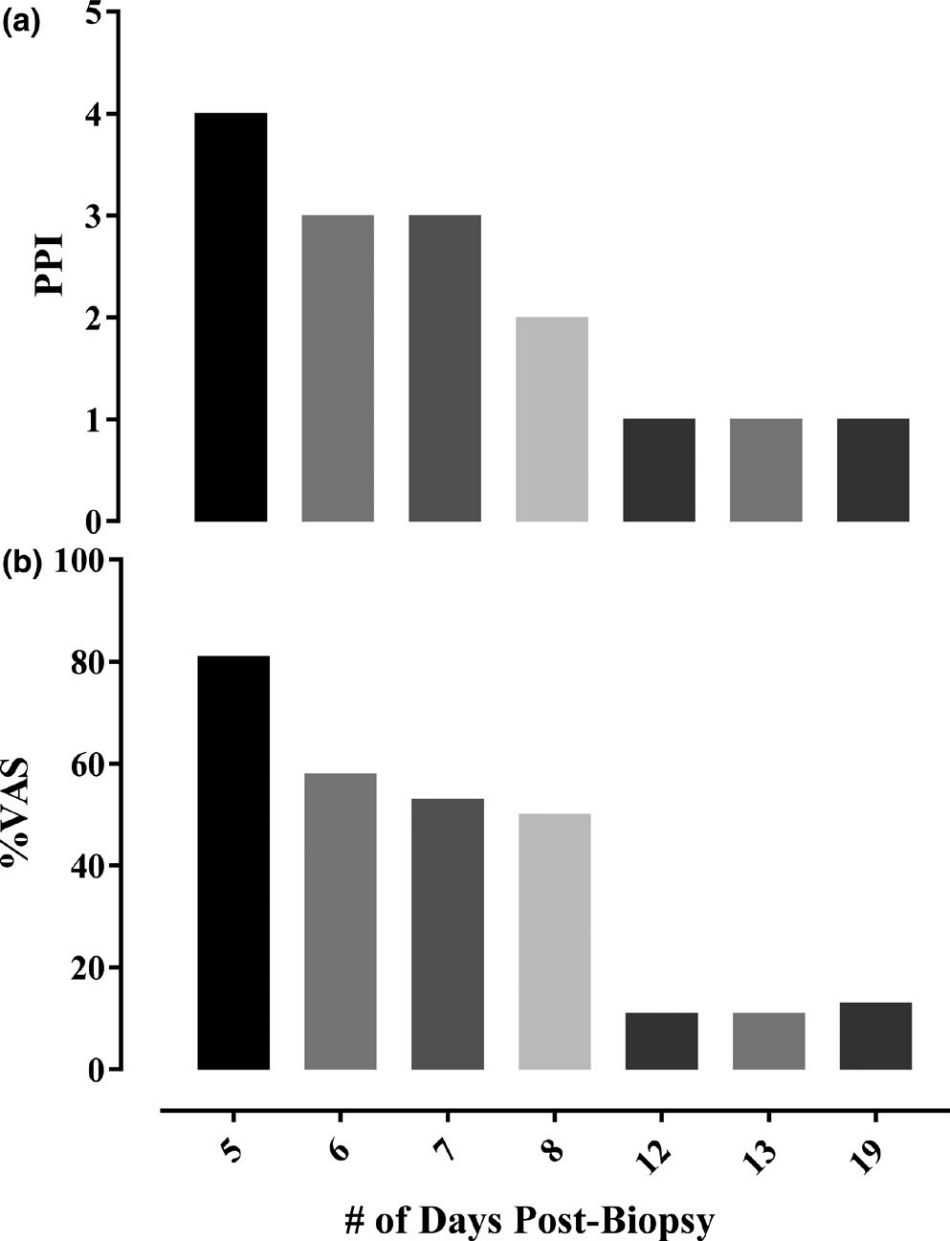
Present pain inventory (PPI; a) and intensity of pain (b) assessed using a visual analog scale (VAS) as part of the short‐form McGill Pain Questionnaire. 0% = no pain, 100% = worst possible pain

*Day 6*–*8*. On day 6, ultrasonography confirmed pulsatile flow of the heterogeneous mass in the right vastus lateralis (Figure 1b). The same day, the participant was referred to athletic therapy—a thorough assessment was completed but, due to severe tenderness at the site of injury, manipulation was deemed unfeasible. The athletic therapist recommended passive stretching, compression, and ice. By day 8, pulsatile flow ceased, and the heterogeneous mass became stagnant (Figure 1c). Along with the absence of pulsatile flow, reported pain also decreased (Figure 2). Despite the reduced pain, the participant maintained an antalgic gait pattern and was unable to participate in any physical activity more intense than walking.

### 3.3 Nonpulsatile period

*Day 12*–*19*. On day 12 and 13 pain was further reduced (Figure 2 and Table 1), however, the site of incision remained tender, achy and the participant reported throbbing pain. The participant's antalgic gait pattern was now reduced, though still present, and he remained unable to participate in athletic activities. On day 19, pain remained unchanged (Figure 2, Table 1), however, the participant attempted to participate in beach volleyball where he reported tolerable pain while running.

**TABLE 1:** Type and severity of pain experienced over the course of recovery from muscle biopsy‐induced artery hemorrhage

		Day post‐biopsy						
		Pulsatile Period				Nonpulsatile Period		
		Day 5	Day 6	Day 7	Day 8	Day 12	Day 13	Day 19
Descriptor	Throbbing	3	2	2	2	1		1
	Shooting	3	1	1	1			
	Stabbing	3						
	Sharp	3	1	1	2			
	Cramping	2			1			
	Gnawing				1			
	Hot‐Burning			1				
	Aching	3	2	2	2	1	1	1
	Tender	3	2	3	2	1	1	1

Ratings: 0 (empty cell) = none, 1 = mild, 2 = moderate, 3 = severe.

*Day 28*–*29*. On day 28, the participant had a referral to a clinical ultrasound at 08:30 and met with the orthopaedic surgeon on day 29. The radiologist, reported a heterogeneous mass within the deeper aspect of the right vastus lateralis measuring 3.1 × 4.3 × 1 cm. This same ultrasound demonstrated a vessel extending around the periphery of the heterogenous mass. The orthopaedic surgeon saw no threat to the heterogenous mass and recommended no action. At this time, the participant reported no pain. The participant had recommenced participation in all athletic activities.

## 4 CASE INTERPRETATION

We interpret the data to suggest that the participant suffered from a muscle biopsy‐induced hemorrhage of an artery in the right vastus lateralis—pulsatile flow in the heterogenous mass supports this interpretation (Figure 1b). Following day 8 the hemorrhage closed thereby inhibiting further arterial inflow. Consequently, outflow was also inhibited thereby trapping blood intramuscularly leading to an intramuscular hematoma which persisted for another 20+ days.

## 5 DISCUSSION

Muscle biopsy adverse advents are infrequent and unpredictable (Tarnopolsky et al., 2011). Although utmost care is taken to avoid major vessels and nerves of the lower limb, it is difficult to always avoid smaller vessels or nerves. In the event a smaller vessel or nerve is hit, there are negative consequences for the participant. Unfortunately, to date, there is limited information about (1) the recovery period following muscle biopsy adverse events and (2) possible methods to minimize risk of percutaneous muscle biopsy adverse events. To help shed some light on one of the possible muscle biopsy adverse events, we have followed the time course of recovery for an individual living with a percutaneous muscle biopsy‐induced hemorrhage/hematoma.

### 5.1 Pain

The discomfort associated with the muscle biopsy‐induced hemorrhage/hematoma was most severe for the participant while the heterogeneous mass was pulsatile (Table 1). During the pulsatile period (Day 0–8), pain was described most as throbbing, aching, and tender with periods of sharp and shooting pain (see Table 1). Following the pulsatile period (nonpulsatile period = Day 9+) pain dropped (Figure 2) and was described as throbbing, aching, and tender. Sharp and shooting pain was absent during the nonpulsatile period (Table 1). Despite a recommendation of 400 mg q4h prn (i.e., every 4 h when required), the participant took 800 mg ibuprofen following both instances of severe, sharp, and shooting pain. The anti‐coagulant effect of the high ibuprofen dose may have contributed to the continued bleeding—this could not be confirmed.

### 5.2 Physical activity

During the pulsatile phase, any physical activity requiring the lower limb was severely limited—the participant was limited to walking on flat surfaces. Despite the reduced pain during the nonpulsatile period, physical activity remained challenging for 20 days post‐cessation of the pulsatile flow. It is likely that the hematoma led to increased intramuscular pressure, making leg flexion more difficult and uncomfortable.

### 5.3 No chronic consequences

Twenty‐eight days after the muscle biopsy‐induced hemorrhage/hematoma the participant was back to his normal routine. A 1 year follow‐up was conducted—the participant reported no noticeable limitations and the hematoma was no longer noticeable via ultrasonography. Therefore, there appears to be no chronic consequences of this muscle biopsy.

### 5.4 Muscle biopsy adverse event risk mitigation

The prevalence of muscle biopsy‐induced hematoma via the Bergstrom technique is low at 1 in 6813 (Tarnopolsky et al., 2011). We have completed <300 percutaneous muscle biopsies and here we report a muscle biopsy‐induced hematoma via the percutaneous technique—raising concern over the use of the percutaneous biopsy technique. Furthermore, considering the small muscle biopsy sample—when compared to the Bergstrom technique—the percutaneous biopsy technique is not useful for comprehensive histological and molecular assessment. Despite being perceived as less painful compared to the Bergstrom technique (Bonafiglia et al., 2020), continued use of the percutaneous muscle biopsy technique should be met with caution.

To help mitigate muscle biopsy‐mediated adverse events, we recommend that compression of the biopsy site be completed for up to 15 min (Thore et al., 2001) immediately post‐biopsy needle removal—minimizing bleeding and hematoma development. Participants should be monitored for up to 72 h, paying close attention for swelling, increased pain, extension of tenderness from the biopsy site, and prolonged restricted range of motion (Smith et al., 2006). These symptoms would indicate the development of a hematoma—exercise should be discouraged until the participant can move their limb while respecting their pain (Smith et al., 2006). Additionally, while the mass is pulsatile, compression should be applied to minimize the size of the developed hematoma (Thore et al., 2001).

## 6 CONCLUSION AND KEY CLINICAL MESSAGES

Although the microbiopsy technique makes smaller cuts in the muscle compared to the Bergstrom technique, there remains a risk of unintentional damage to neighboring vessels or nerves. The present case report demonstrates that the inadvertent hemorrhaging of a neighboring vessel can be debilitating. The recovery of the hemorrhaged vessel can be rapid, though the recovery from the remaining intramuscular hematoma can take much longer. Although chronic consequences do not appear to be evident, the acute consequences were severe for this individual and therefore risk minimization for future participants is warranted. To minimize the risk of muscle biopsy‐induced hemorrhage/hematoma, we advise post‐biopsy compression for up to 15 min and careful post‐biopsy follow‐up should be completed for up to 72 h—paying attention for swelling, increased pain, extension of tenderness from the biopsy site, and prolonged restricted range of motion (Smith et al., 2006). When any of these symptoms are evident, the participant should avoid exercise and compression should be applied while the mass is pulsatile—minimizing the size of the developed hematoma.

## CONFLICTS OF INTEREST

No conflicts of interest, financial or otherwise, are declared by the authors.

## AUTHORS’ CONTRIBUTIONS

P.J.D, H.I, C.A.S, and B.J.G, Conceived and designed research, P.J.D completed patient interview, P.J.D and H.I collected self‐reported pain questionnaires, P.J.D, H.I, C.A.S, and.J.G interpreted the case P.J.D synthesized all data, P.J.D prepared figures drafted manuscript, P.J.D, H.I, C.A.S, and B.J.G edited and revised manuscript, P.J.D, H.I, C.S, and B.J.G approved final version of manuscript.

## CONSENT FOR PUBLICATION

Written informed consent was obtained from the patient for publication of this case report and any accompanying images.

## ETHICS APPROVAL AND CONSENT TO PARTICIPATE

Experimental procedures for both the original study the participant enrolled in and the current report were approved by the Health Sciences Human Research Ethics Board at Queen's University in accordance with the Declaration of Helsinki. The participant was provided with verbal and written explanation of experimental procedures and associated risks prior to giving written informed consent.

## Data Availability

The datasets generated and/or analyzed during the current study are available from the corresponding author on reasonable request.
